# An Open-Source COVID-19 CT Dataset with Automatic Lung Tissue Classification for Radiomics

**DOI:** 10.3390/bioengineering8020026

**Published:** 2021-02-16

**Authors:** Paolo Zaffino, Aldo Marzullo, Sara Moccia, Francesco Calimeri, Elena De Momi, Bernardo Bertucci, Pier Paolo Arcuri, Maria Francesca Spadea

**Affiliations:** 1 Department of Experimental and Clinical Medicine, University “Magna Graecia” of Catanzaro, 88100 Catanzaro, Italy; mfspadea@unicz.it; 2 Department of Mathematics and Computer Science, University of Calabria, 87036 Rende, Italy; marzullo@mat.unical.it (A.M.); calimeri@mat.unical.it (F.C.); 3 Department of Information Engineering, Università Politecnica delle Marche, 60131 Ancona, Italy; s.moccia@staff.univpm.it; 4 Department of Advanced Robotics, Istituito Italiano di Tecnologia, 16163 Genova, Italy; 5 Department of Electronics, Information and Bioengineering (DEIB), Politecnico di Milano, 20133 Milano, Italy; elena.demomi@polimi.it; 6 Department of Radiology, Pugliese-Ciaccio Hospital, 88100 Catanzaro, Italy; bernardo.bertucci118@gmail.com (B.B.); arppaolo@alice.it (P.P.A.)

**Keywords:** COVID-19, free CT dataset, medical imaging, radiomics

## Abstract

The coronavirus disease 19 (COVID-19) pandemic is having a dramatic impact on society and healthcare systems. In this complex scenario, lung computerized tomography (CT) may play an important prognostic role. However, datasets released so far present limitations that hamper the development of tools for quantitative analysis. In this paper, we present an open-source lung CT dataset comprising information on 50 COVID-19-positive patients. The CT volumes are provided along with (i) an automatic threshold-based annotation obtained with a Gaussian mixture model (GMM) and (ii) a scoring provided by an expert radiologist. This score was found to significantly correlate with the presence of ground glass opacities and the consolidation found with GMM. The dataset is freely available in an ITK-based file format under the CC BY-NC 4.0 license. The code for GMM fitting is publicly available, as well. We believe that our dataset will provide a unique opportunity for researchers working in the field of medical image analysis, and hope that its release will lay the foundations for the successfully implementation of algorithms to support clinicians in facing the COVID-19 pandemic.

## 1. Introduction

The coronavirus disease 19 (COVID-19) is an infectious respiratory disease caused by a strain of the coronavirus SARS-CoV-2, which was first identified in Wuhan, China in December 2019. The disease mainly affects airways and lungs with the onset of pneumonia and acute respiratory distress syndrome.

The response to the disease can be very different, ranging from asymptomatic conditions to more severe ones. Studies in the literature have agreed on the diagnostic role of reverse transcriptase-polymerase chain reaction (RT-PCR), the accepted standard until now. However, since the pandemic’s onset, imaging has played an important role for prognosis and follow up [1,2]. So far, computed tomography (CT) findings in the chest were not shown to be specific for COVID-19 [2]. CT images can rather be used to evaluate the presence and extent of opacifications, known as ground glass opacities (GGOs), and consolidations. To date, such evaluations are mainly qualitatively performed, posing issues that are relevant to their repeatability and reliability. Researchers working in the field of medical image analysis are trying to attenuate these issues by developing algorithms for automatically scoring the pulmonary involvement in COVID-19 pneumonia. In such a framework, collecting and sharing imaging datasets are more crucial than ever [3]. In this scenario, the main problem is that public datasets are often made up of a small number of patients and are provided as low-quality images. On the other hand, several institutions that own large collections of high-quality scans are often not inclined to anonymously share the data. As a result, in spite of some recent efforts in the literature [4,5], public COVID-19 CT datasets are still mostly limited in size and quality [6,7,8]. The lack of large and high-quality datasets in the field hampers the development and translation of the medical image analysis methodology developed in actual clinical practice. This is a strong issue, considering that we are still facing the pandemic and that the clinical community is asking for computer-assisted solutions to provide clinicians with decision support. In this work, we try to attenuate this issue by presenting a labeled high-quality dataset of 62 CT volumes, consisting, on average, of 827 slices (min–max range: 248–1251) for a total of 51,247 slices acquired from 50 COVID-19-positive patients. The CT volumes are provided along with an automatic lung tissue annotation, as well as with a scoring provided by an expert radiologist, ranging from 0 (clinically healthy) to 5 (pathological), in unit increments. This score was found to correlate with the presence of GGOs and automatically predicted consolidation. We hope that the COVID-19 CT dataset will stimulate research in the field to help clinicians in the fight against the pandemic; for instance, possible uses of the proposed integrated dataset include the development of strategies for stratification and radiomic analysis of patients for a better insight into this pathology.

The rest of this article is structured as follows: In Section 2, the details about the proposed images and the label generation strategy are reported; in Section 3, the label validation is assessed; in Section 4, the presented dataset is discussed. Finally, Section 5 presents our conclusions.

## 2. Materials and Methods

### 2.1. Data Acquisition

**Figure 1:**
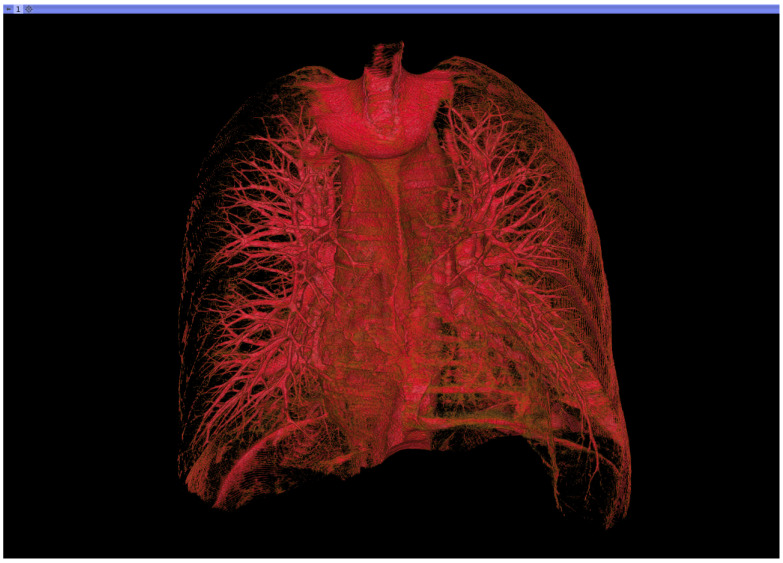
Exemplary 3D rendering of coronavirus disease 19 (COVID-19)-affected lungs. The clinical score *S* for the depicted patient is equal to 3. In the inferior left lobe, it is possible to see some opacities due to COVID-19.

The dataset includes the images of 50 COVID-19 patients (diagnosis confirmed by a positive RT-PCR test) who received a non-contrast chest CT at Azienda Ospedaliera Pugliese-Ciaccio (Catanzaro, Italy) with reconstructions of the volume at 0.3 to 1 mm slice thickness. The patients’ average age was 56 years (range 20–83), and the male/female ratio was 23/27. Images were obtained with two different scanners present at the clinical facility: (1) Siemens Somatom Go.now (Siemens Healthcare GmbH, Erlangen, Germany) and (2) Toshiba Aquilion ONE (Canon Medical System Europe B.V., Zoetermeer, The Netherlands). A total of 38 patients were scanned once only for diagnostic purposes, while CT images of 12 patients were also acquired for follow up. So, a total of 62 CT volumes are available in the database. A summary of the proposed dataset is reported in Table 1, while an exemplary 3D rendering of COVID-19-affected lungs is shown in Figure 1.

In order to facilitate data sharing and processing, the DICOM files were anonymized and converted into nrrd (http://teem.sourceforge.net/nrrd/ (accessed on 15 February 2021) files by using 3D Slicer [9,10].

The image collection reported here was conducted with approval from the Hospital Ethics Committee, “Prot. 308”.

### 2.2. Visual Assessment

The primary findings concerning COVID-19 in CT images are those of atypical pneumonia [11] and include two macroscopic lung tissue abnormalities: ground glass opacity (GGO), i.e., an area of increased X-ray attenuation in the lung with preserved bronchial and vascular markings, and consolidation, which refers to the filling of the pulmonary tree with material that attenuates X-rays more than the surrounding lung parenchyma.

With these premises, an expert radiologist (with more than 20 years in the field) was asked to visually evaluate the CT images and provide a clinical score, namely *S*, based on the extent of lung involvement with the above-mentioned manifestations. The radiologist navigated through the volume in the axial, sagittal, and coronal planes, assigning a value between 0 and 5 according to the following scale [12]:

0. 0% lung involvement;
1. <5% lung involvement;
2. 5–25% lung involvement;
3. 26–50% lung involvement;
4. 51–75% lung involvement;
5. >75% lung involvement.

This scores are provided in the open dataset together with each image volume.

### 2.3. Automatic Segmentation

The lung tissue classification pipeline proposed in this work consisted of different steps. First, lung region segmentation was performed on each CT image: (i) Threshold-based segmentation was used to divide pixels into two classes (lung and background). The optimal threshold was empirically found to be –155 HU. (ii) Small-island removal was performed to discard possible non-lung clusters that survived the thresholding (e.g., intestines). The minimum number of voxels included in an island to be removed was set to $5*10^{5}$. (iii) Pixels classified as lung were processed using morphological closing filters in order to fill the remaining holes in the region of interest. The segmentation was used to extract only the relevant region of interest inside the scan, discarding all the unnecessary information. Then, images were represented as a one-dimensional array of voxels and stacked together with the others to form a single one-dimensional array. The obtained array was used to fit a five-component Gaussian mixture model (GMM) (scikit learn implementation, six different initializations based on the kmeans algorithm with a convergence threshold equal to $1*10^{-3}$) in order to detect air, healthy lungs, GGOs, consolidations, and other denser tissues (including vessels, bronchi, and fibrotic stripes). These classes refer to healthy and pathological tissues that can be detected in CT images for clinical evaluation. Finally, for each CT image, the lung voxels were labeled with the most probable component with a maximum a posteriori probability (MAP) estimate.

**Figure 2:**
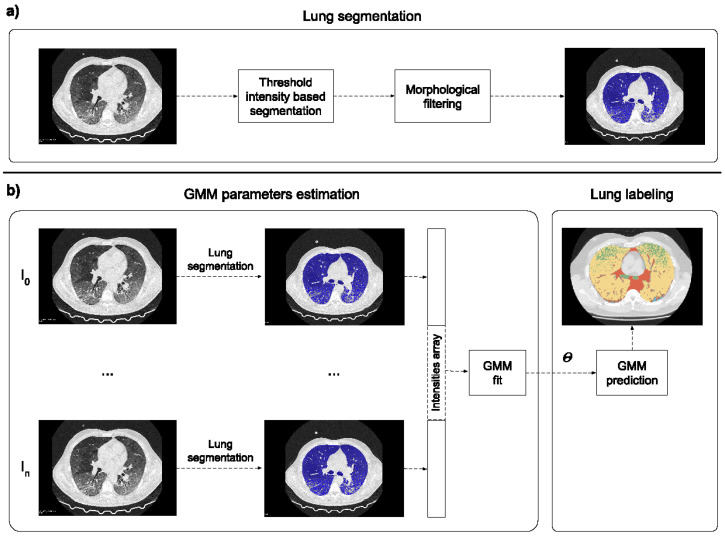
Proposed labeling pipeline split into lung segmentation (panel (**a**)) and tissue labeling (panel (**b**)). After lung region segmentation (performed by using the worklflow in (**a**)), lung voxels of different computed tomographies (CTs) were represented as a single one-dimensional array used to fit a five-component Gaussian mixture model (GMM). Once the algorithm converged, each CT image was labeled using the estimated parameters (θ).

The whole pipeline is represented in Figure 2.

**Figure 3:**
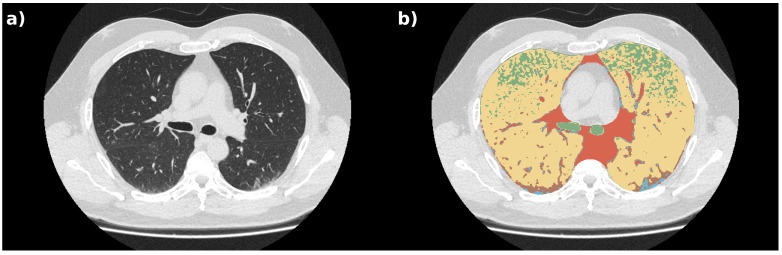
Exemplary axial view of an anatomical image (panel (**a**)) and labeled volume (panel (**b**)) extracted from patient 17 (S = 1). The green label represents air, the yellow label marks healthy lungs, the light blue label indicates ground glass opacity (GGO), brown voxels are consolidations, and orange clusters are other denser tissue.

Tissue labels obtained with the GMM were processed by a median filter [13] (scipy implementation, sigma equal to 4) in order to filter out spurious pixels. This smoother segmentation is available in the proposed database alongside the voxel-per-voxel classification. This latter might be useful for radiomics applications. A median filter was applied to generate label maps that generally classified areas rather than specific single voxels. An exemplary case (both anatomical and label volumes) is depicted in Figure 3.

Due to the lack of ground-truth segmentation, the automatic labeling was assessed in terms of the robustness of the unsupervised labeling method and the correlation with clinical scoring. In the first test, the robustness of the GMM prediction was assessed by evaluating the fluctuation of model parameters computed by using different combinations and numbers of patients included for fitting; in the second test, the correlation between the automatic segmentation and clinical condition was verified.

### 2.4. Presented Dataset

The dataset presented herein is composed of a homogeneous set of anatomical images alongside clinical scores and automatic lung tissue labeling.

At the time of writing, it comprises 62 instances from 50 patients. However, we aim to create a dynamic dataset, rather than a static collection, adding patients when new scans become available.

No external data records are included.

All images are provided in the same format: The choice of this file format was made with the manifold aim of guaranteeing complete anonymization, simplifying data sharing, and maximizing compatibility with research software. The project is intended to provide the community with a valuable source of high-quality data for further research; hence, the dataset presented herein is released and licensed under CC BY-NC 4.0 (https://creativecommons.org/licenses/by-nc/4.0/ (accessed on 15 February 2021)). As stated by the license itself, the dataset is open and the use is free and encouraged, though any commercial use is forbidden.

The dataset can be accessed at https://www.imagenglab.com/newsite/covid-19 (accessed on 15 February 2021).

The code for identifying GMM parameters and executing patient segmentations is freely available at https://github.com/pzaffino/COVID19-intensity-labeling (accessed on 15 February 2021). The precomputed GMM model was uploaded in the same repository as well. Label generation was performed by using Python 3.8.5, numpy 1.19.2, scipy 1.5.2, SimpleITK 1.2.4, scikit learn 0.23.2, scikit image 0.17.2, and joblib 0.16.0. In its current state, the code is intended for research purposes only, and it was not designed to be used in a real clinical scenario.

## 3. Results

**Figure 4:**
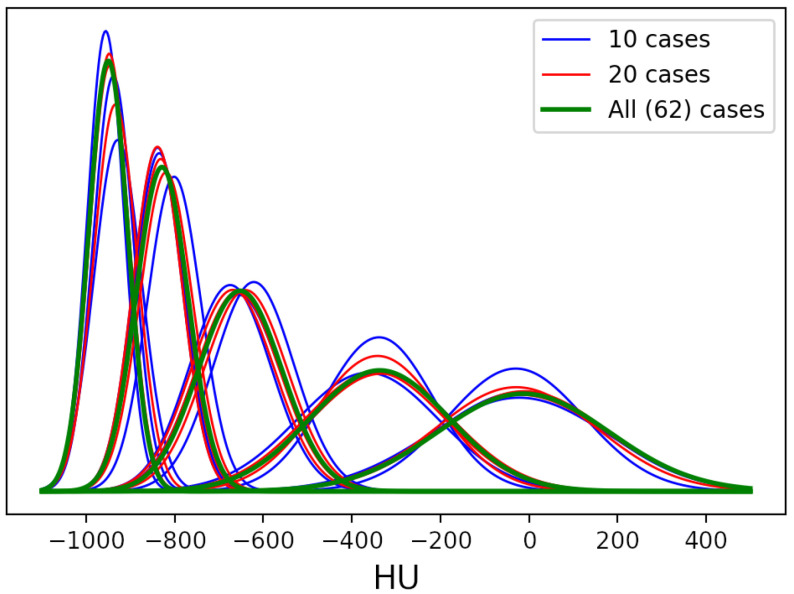
Estimated Gaussians for each fold of the robustness test. Multiple lines of the same color show the results obtained from different groups of the same fold size.

The robustness of the unsupervised labeling method was assessed by randomly subsampling the dataset in a series of folds of increasing size; in particular, we started from a fold of size 10 with steps of 10. In order to take noise and random effects into account, we randomly extracted three different groups for each fold size. The stopping criterion was the “convergence” of GMM parameters, i.e., stability. One can observe that the distribution already tends to overlap among different groups with a size of 20, as shown in Figure 4, which depicts the estimated Gaussians.

In order to assess the clinical significance of the automatic tissue labeling, the lung involvement (LI) was measured using:

(1) LI=%GGO+%consolidation+%other_tissues,

where *%GGO* is the percentage of ground glass opacities in the lung volume, *%consolidation* is the fraction of consolidation, and *%other_tissues* is the percentage of the identified denser tissue. The Pearson correlation coefficient, C, between S and LI was computed, resulting in 0.82 (*p*-value $<10^{-6}$).

**Figure 5:**
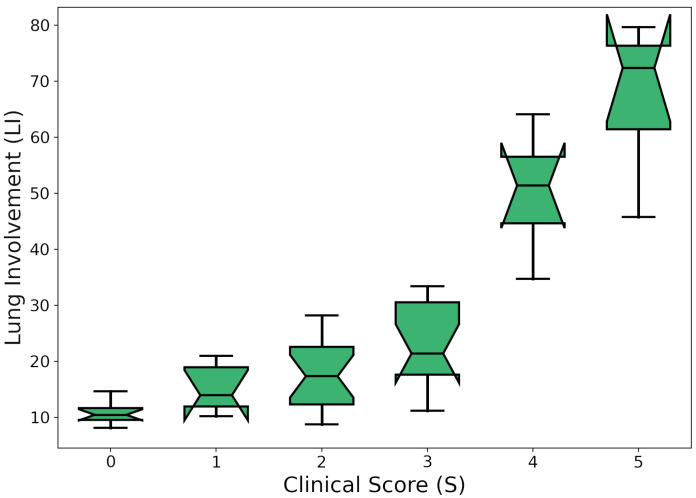
Box plot of S vs. lung involvement (LI). For each S, the median ± quartiles and min–max of LI are reported.

Figure 5 shows the box plot and the scatter plot of S vs. LI.

The coefficient of determination ($R^{2}$) was estimated to assess the proportion of the variance in the dependent variable (scores) that is predictable from the independent variable (CT coefficients). It is worth noting that the considered dataset is highly unbalanced with respect to S = 2 and S = 3. This is expected for two reasons: (1) Most of the patients arrived at the hospital with pneumonia symptoms, but very few were critical; (2) uncertain evaluations are likely to fall into the central categories. In order to take the problem into account, an iterative resampling strategy was carried out. In particular, for n = 100 iterations, *R* and $R^{2}$ were computed by fitting a linear regression model, drawing a proportional number of samples from each group in the original population. Mean ± standard deviation values for $R^{2}$ and *C* were 0.72±0.03 and 0.89±0.01, respectively. As a result, the clinical score was found to strongly correlate with the presence of GGOs and consolidations predicted by the GMM.

## 4. Discussion

In this paper, an open dataset of COVID-19 CT images was presented. In addition to anatomical images, automatic tissue labeling and clinical scores were provided as well. The CTs had a sub-millimetric slice thickness and, unlike other publicly available datasets, volumes are provided in an nrrd file, one of the most common ITK-based [14] formats. In this way, both intensities and geometrical information are fully preserved, thus guaranteeing, at the same time, full data anonymization and practical file management. To the best of our knowledge, this is one of the first free, public, and high-resolution labeled datasets that also includes clinical information (such as a radiologist’s score and patients’ genders). As a result, accurate, advanced, and quantitative analyses can be conducted. Examples of free and open-source solutions that are able to natively support the format used to store the data are 3D Slicer [9,10] (visualization and processing), ITK-SNAP [15] (visualization), Plastimatch [16] (image registration and processing), and ITK [14]/SimpleITK [17] (complete toolkit for medical image processing). Pyradiomics [18], for instance, could be used to run radiomics analyses on the proposed dataset, either as a standalone tool or as a 3D Slicer plugin.

According to the permission of the ethics committee and the chosen license (CC BY-NC 4.0), scientific usage of the dataset is encouraged, while any commercial use is forbidden. Each CT is made of about a hundred of slices, so manual labeling is practically unfeasible. In addition to the aspect of time consumption, it is very difficult for a radiologist to assign a well-defined tissue class to each lung voxel. For these reasons, relying on the assumption that different tissue classes have different densities, a machine learning approach was chosen to cluster voxels based on the probability of being part of a certain class. As a consequence, shape, anatomical, and spatial information was not included in the process. While this strategy allows one to classify tissues without a priori information (such as manual contours), in some cases, some voxels can be erroneously labeled. Additional errors could caused by the intensity threshold segmentation and the series of morphological filters used to identify the lung tissues. Although this is a key step for a completely automatic workflow, occasionally, it can include some non-lung tissues (e.g., portions of the heart and/or vertebrae) and/or exclude some portions of the volume of interest (such as part of the fibrous stripes close to the lung wall). In order to mitigate this problem, a class named “non-lung tissue” was added to the classification process.

Due to the lack of ground-truth volumes, the automatic contours were validated by computing the correlation between the amount of automatically identified pathological tissues and the clinical score provided by an expert radiologist. The obtained correlation value confirmed our initial hypothesis. The machine learning algorithm convergence was assessed as well.

Finally, one of the strengths of the proposed labeling strategy, unlike other available datasets, is the possibility to independently classify each voxel rather a group of voxels (as in manual or deep-learning-based segmentations). In this way, it could be possible to extract some quantitative radiomic features that could help to unveil hidden connections between imaging and clinical conditions.

## 5. Conclusions

In conclusion, a free COVID-19 CT dataset made of 62 high-resolution volumes was presented for research purposes. Alongside the anatomical images, clinical assessments of the severity of the disease and automatic lung tissue labeling were provided as well. The code for reproducing the experiments and for labeling unseen patients is also publicly available.

## Figures and Tables

**Table 1:** Dataset characteristics.

Number of CTs	Scanner	Slice Thickness [mm]	Kilovoltage Peak
46	Siemens Somatom Go.now	0.3 (2 cases 1.0)	110
16	Toshiba Aquilion ONE	0.4	120

## Data Availability

The data presented in this study are available under the CC BY-NC 4.0 license at https://www.imagenglab.com/newsite/covid-19/.
